# ncRNAs in breast milk of mothers with SLE: potential implications for neonatal immune development

**DOI:** 10.3389/fimmu.2026.1840082

**Published:** 2026-09-01

**Authors:** Julia Wiktoria Chwatko, Aizhan Rakhmetullina, Piotr Zielenkiewicz, Leszek Pączek

**Affiliations:** 1 Clinical Immunology Student Scientific Association, Medical University of Warsaw, Warsaw, Poland; 2 Institute of Biochemistry and Biophysics, Polish Academy of Sciences, Warsaw, Poland; 3 Institute of Experimental Plant Biology and Biotechnology, University of Warsaw, Warsaw, Poland; 4 Department of Clinical Immunology, Medical University of Warsaw, Warsaw, Poland

**Keywords:** breast milk, lncRNA, miRNA, ncRNA, neonatal development, SLE, systemic lupus erythematosus

## Abstract

Systemic lupus erythematosus (SLE) is an autoimmune disease primarily characterized by the production of autoantibodies that induce tissue damage. Although the etiology of the disease remains unclear and may be attributed to numerous factors, various studies emphasize the role of epigenetic mechanisms in SLE. In particular, alterations in non-coding RNA (ncRNA) expression have been identified as potential causes as well as promising biomarkers of the disorder. ncRNAs are a diverse group of molecules involved in the regulation of gene expression. Due to their accessibility in bodily fluids, such as serum or breast milk, they are considered promising biomarkers for different diseases. As SLE is more common among adult women of reproductive age, this raises the question of whether alterations in ncRNA levels in the breast milk of mothers with SLE might affect the breastfed infant. Breast milk is essential for the development of the immune response in infants; therefore, it is important to identify alterations in ncRNAs that may be relevant for neonatal development. Although there is currently no direct evidence of altered ncRNA levels in the breast milk from mothers with SLE, this literature review aims to examine various ncRNA molecules associated with SLE in terms of their presence in human breast milk and their impact on the neonatal immune system.

## 1 Introduction

Systemic lupus erythematosus (SLE) is a complex and multifactorial autoimmune disease. The etiology of the disease is unclear. Speculations regard environmental, ecological, epigenetic, and genetic factors as variables potentially contributing to the onset of SLE (1). In terms of its pathogenesis, SLE is primarily associated with enhanced production of autoantibodies against nuclear and cytoplasmic antigens by B-cells, resulting in damage to tissues and organs (1). One of the hypotheses concerning the pathogenesis of SLE perceives molecules such as non-coding RNAs (ncRNAs), as potential regulators of the disease. To illustrate, they are suggested to dysregulate B-cell activation through signaling pathways linked to nuclear factor kappa light chain enhancer of activated B cells (NF-κB), type I interferons (IFN-I), and transforming growth factor (TGF) (2). Owing to the heterogeneity in presentation of the disease, its clinical manifestations generally include skin manifestations, arthritis, and hematological and immunological symptoms (1). However, it may also affect numerous other systems. In regard to the epidemiology of SLE, it is more commonly encountered in adult women of reproductive age (1).

Due to their vast diversity in shape, location, and length, ncRNAs can be divided into classes – microRNA (miRNA), long ncRNA (lncRNA), circular RNA (circRNA), and PIWI-interacting RNA (piRNA). In terms of their length, miRNAs are small RNA molecules with approximately 22 nucleotides, whereas lncRNAs and circRNAs are composed of more than 200 nucleotides (3). Moreover, the function of miRNAs is to degrade the targeted mRNA. It is achieved through binding to a complementary sequence in the mRNA molecule and subsequently forming an RNA-induced silencing complex (RISC). In comparison to miRNA, lncRNAs and circRNAs also control gene expression; however, they may prevent the degradation of mRNA by acting as a miRNA decoy (3). Consequently, altered expression of ncRNAs in T-cells might contribute to immunodeficiency and eventually lead to the development of autoimmune disease. Thus, they have found application as potential biomarkers for numerous diseases, including SLE (2).

Breast milk is a biofluid that is essential for newborns in terms of nutrition. In addition, it has been discovered that breast milk may also regulate and support the development of their immune systems. Milk generally consists of three fractions - cellular, fat, and skim components (4). Furthermore, it is also rich in microorganisms, immunoglobulins, cytokines and miRNAs, which stimulate the first immune responses in an infant (5). To exemplify, let-7a-5p, which is abundant in breast milk, may contribute to the infant immune response, as it has been identified as a crucial regulator of immune response in general (4). The absorption of miRNA molecules is deemed to be associated with the endocytosis of exosomal vesicles (4). Additionally, studies have shown that this transport is temperature-dependent, as miRNA uptake was decreased when the temperature of milk was reduced from 37 °C, the physiological temperature of the human body, to 4 °C (4), thus suggesting the importance of direct transfer between individuals. Furthermore, an experiment revealing bovine milk miRNAs in human plasma after a meal demonstrated that breastmilk miRNAs could be exchanged between species through the digestive system (4), once again implying that the mechanism of breastfeeding is crucial to ncRNA transfer. Apart from miRNAs, breast milk also contains other regulatory ncRNAs, such as lncRNAs, siRNAs, piRNAs, circRNAs and fragmented tRNAs (6), therefore the need to study the transfer of ncRNAs does not pertain only to miRNAs.

Although peripheral to the main topic, neonatal lupus erythematosus (NLE) is an acquired autoimmune disease in infants. In general, it is induced by the passive transplacental transfer of maternal IgG antibodies against the RNA protein complex - anti-Ro/SSA and anti-La/SSB. The main clinical manifestations of NLE mostly involve cutaneous lesions; however, it also poses a risk of congenital heart block, a condition that may be fatal to the newborns (7). Even though NLE is mainly linked to vertical transmission, there is a scarce number of publications addressing the transmission of antibodies through breast milk. Conceivably, this may be due to the rarity of the condition and its usual transplacental mode of transmission. Nevertheless, one case of NLE presumably related to the transmission of antibodies through breast milk has been reported. The breastfed male infant was noted to have NLE skin lesions. The cause of these lesions was assumed to be linked to the high levels of IgG and IgA in the mother’s milk (8). Moreover, those antibodies were found to be reactive against nuclear and Ro targets. Three weeks after the discontinuation of breastfeeding, the lesions resolved (8). Nonetheless, the overall number of sources on this topic remains limited.

Breastfeeding is proven to be essential for the proper development of the infant immune system; however, the topic of ncRNA in breast milk has not yet been fully explored. Only some ncRNAs have been identified in breast milk, and only some of their functions have been characterized. Furthermore, most studies have focused on miRNAs present in breast milk from presumptively healthy women. Therefore, the question arises as to whether a mother’s pathologically altered immune system may have long-term implications for a breastfed newborn. For example, mothers with SLE may have altered ncRNA levels, which may in turn affect ncRNA content in breast milk. The pattern of dysregulation does not have to be the same for every molecule. For some ncRNAs, their deficiency might be considered a relevant factor in the developing immune system of an infant, whereas for others, their overexpression could have biological consequences, such as enhanced inflammatory processes. According to studies examining breastfeeding rates among mothers with SLE, at least half of the women included in the 2016 and 2021 studies expressed a desire to breastfeed (9, 10). However, the focus of those studies referred mainly to the safety of SLE pharmacotherapy for breastfed infants. To the best of our knowledge, the topic of potential implications for newborns breastfed by mothers with SLE has not yet been thoroughly investigated. This narrative review summarizes the available information on this topic with particular emphasis on miRNAs, given the limited number of available studies. Furthermore, this review explores the plausible role of ncRNAs in linking SLE dysregulation, breastfeeding, and neonatal development. Considering the limited evidence on lncRNA in breast milk and neonatal development, the section concerning lncRNAs is covered only briefly. To collect data, a targeted literature search in PubMed and Google Scholar was performed. Moreover, Nature Portfolio was also manually screened for relevant publications. The following queries were used: (“ncRNA” OR “miRNA” OR “lncRNA” OR “miR-155” OR “miR-146a” OR “miR-21” OR “miR-181a” OR “let-7” OR “miR-210” OR “miR-125b” OR “miR-29a” OR “NEAT1” OR “TUG1” OR “GAS5”) AND (“systemic lupus erythematosus” OR “SLE”) OR (“breast milk” OR “milk-derived” OR “neonatal development” OR “newborns” OR “infants”). From the retrieved results, only studies investigating ncRNAs in SLE, breast milk, or neonatal development were selected. Attention was given to recent studies, original research articles, and comprehensive reviews. The review focuses on publications from 2004 to 2026.

## 2 miRNAs in pathogenesis of SLE

As mentioned above, the pathogenesis of SLE has been investigated from numerous perspectives, such as genomic, epigenomic, transcriptomic, or metabolomic. From an epigenomic perspective, dysregulated ncRNAs, mainly miRNAs, are of particular interest because of their crucial role in the immune system (11). Although the exact function of each molecule has yet to be confirmed, their potential as future biomarkers and therapeutic targets is being actively studied. To illustrate, miR-146a has been linked to the disease in terms of its activity in IFN and NF-κB inflammatory pathways, thereby regulating SLE-associated genes, such as *TRAF6*, *STAT1*, *IRF5*, and *IRAK1* (11). With regard to disruption of DNA methylation, miRNAs regulate the mRNA expression of methyltransferases, for instance *DNMT1*, *DNMT3A*, and *DNMT3B*. For example, miR-29b and miR-126 lead to decreased *DNMT1* levels in T-cells. Additionally, the overexpression of miR-126 is also linked to the upregulation of CD11a and CD70, which contribute to T-cell and B-cell hyperactivity (12). Shifting the focus to the B cells, their growth and differentiation are strongly affected by cytokines, whereas cytokine overproduction is related to one of the mechanisms of SLE. miR-181a was found to correlate with increased expression of pro-inflammatory factors, such as IL-1β, IL-6, IL-8, or TNF-α. Similarly, miR-146a, as part of IRAK1/TRAF6/IKKβ/NF-κB pathway, was also found to be linked to pro-inflammatory cytokine production (12). On the other hand, miR-21 suppresses IL-10 production as well as the proliferation of CD4+ T-cells (12). In addition, its expression in SLE was shown to affect another mechanism – autophagy – by promoting activation of the PI3K/Akt/mTOR pathway. As a result, it caused abnormal B-cell differentiation. Another potential mechanism is related to the proposed influence of miR-223, miR-25, and let-7a on the mammalian target of rapamycin (mTOR) pathway, which also influences autophagy and cytokine production (12, 13).

The role of sex in SLE pathogenesis has yet to be fully elucidated and may help explain the higher prevalence of the disease among women. One hypothesis suggests the involvement of incomplete X chromosome inactivation linked to the lncRNA X inactive-specific transcript (*Xist*) (14). A study by Scofield et al. points to two genes in the Toll-like receptor 7 (TLR7) pathway as candidates escaping X chromosome methylation (14). This process is also linked to the overexpression of miR-98, miR-188-3p, and let-7f-2 on the X chromosome (12). In conclusion, all of these hypotheses indicate that the pathogenesis of SLE is most likely multifactorial and involves numerous molecules. Thus, this review aims to explore some of them, focusing on those most frequently recognized in academic literature, both in the context of SLE and in developmental processes.

## 3 miRNAs in bodily fluids

Research on the role of ncRNAs as potential biomarkers of SLE has mostly focused on bodily fluids, such as serum, plasma or urine. There is little information about changes in the composition of breast milk in patients with SLE. A study by Alsaweed et al. aimed to expand on the link between the physiological content of miRNAs in peripheral blood mononuclear cells (PBMCs) and human milk cells. Their findings indicated some overlap in miRNA composition; however, the miRNA profiles varied. Thus, they concluded that those miRNAs in human milk were endogenously synthesized in lactocytes. On the other hand, the possible influence of maternal circulation on the miRNA content of milk was not excluded (15). Similarly, another study demonstrated rather diverging miRNA profiles between plasma and breast milk. Nevertheless, they found an abundance of let-7f-5p in milk and plasma of about 2.169% and 0.288%, respectively. Additionally, they showed that the most abundant molecule in milk was miR-148a-3p, whereas in plasma it was miR-486-5p (16). According to Muse et al., only nine miRNA species were present both in plasma in the second trimester and in breast milk at 6 weeks postpartum, including miR-320e, miR-1253, and miR-378e. However, this disparity was attributed to different needs during specific developmental windows. Notably, they reported a correlation between miRNA profiles in exosomal vesicles and the time of sample collection, maternal pre-pregnancy weight, mode of delivery, parity, and infant age (17). Overall, the evidence suggests that ncRNA molecules in breast milk and peripheral blood may have different origins. Nevertheless, the possibility that these origins are interlinked has not yet been thoroughly explored.

Mothers who are unable to breastfeed their offspring are offered the various milk-based formulas as an alternative. However, this raises the question of whether those formulas contain all the necessary nutrients, including potentially crucial ncRNAs. Alsaweed et al. compared the miRNA content among formulas. Bovine milk-based formula more closely resembled human milk than soy-based formula; however, its miRNA content was scarce. In both of those artificial formulas miR-159a predominated over other species. The study also reflected on the stability of ncRNAs, which are subjected to industrial processing, during which the fat layer and debris in milk are discarded (15). Taken together, the lack of sufficient evidence suggests a possible need to identify ncRNA molecules significant for neonates and, eventually, to enrich formulas in order to provide children with the necessary nutrients.

## 4 miRNA absorption in infants

Exosomes, extracellular vesicles measuring between 30 to 150 nm in diameter, encapsulate the contents of breast milk, such as miRNAs, mRNAs, proteins, or lipids, and provide stable transport (4, 18). The exact mechanism of miRNA absorption in humans is still unknown. However, studies on bovine milk molecules, such as miR-21-5p and miR-30a-5p, found them to be measurable in human plasma for up to 6 hours after a meal containing bovine milk (4). In addition, further *in vitro* experiments on cell lines have been conducted to provide additional evidence (4). Absorption is presumably linked to SID-1 transmembrane family member 1 (SIDT1), which is responsible for facilitating endocytosis (4). miRNA bioavailability depends on stability against RNase activity, the ability to reach the target site, and the maintenance of a particular concentration sufficient to activate a response (19). The biodistribution of exosomes after uptake is mainly localized in the liver and spleen, with the miRNAs most likely being removed by macrophages (4). However, this topic requires further investigation, as all of these processes remain largely hypothetical.

The gastrointestinal (GI) tract in early infancy is still immature; hence, the breakdown of bioactive molecules may be diminished. This is associated with an increased gastric pH, decreased enzyme activity, as well as reduced barrier integrity, which may in turn be linked to shorter enterocytes and altered tight junction composition (20). Exosomes consumed by neonates are believed to protect against inflammatory processes, particularly those containing miR-148a and miR-30b. Their role may also extend to protection against necrotizing enterocolitis by stimulating regulatory T-cells (Treg cells) differentiation and the growth of *Bifidobacterium* or *Lactobacillus* (18). Some studies also point to their role in microglial plasticity and neurodevelopment through interaction with the DNMT1 enzyme (21).

## 5 The link between miRNAs in SLE and neonatal development

**Figure 1:**
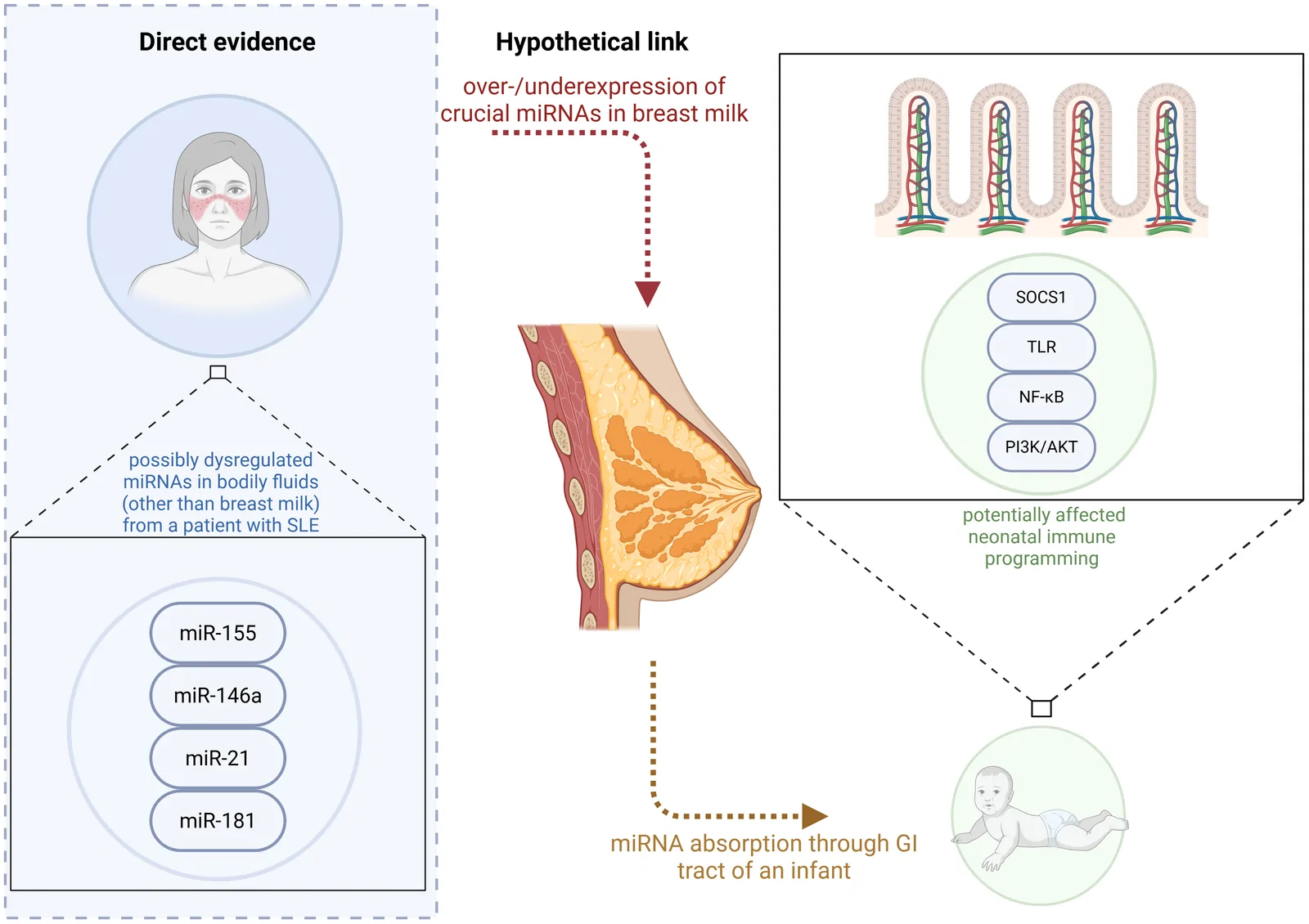
Proposed model of the potential impact of maternal SLE-associated miRNAs on the development of neonatal immune system. Created in BioRender. (2026) https://BioRender.com/6ocmp16.

As stated above, dysregulation of miRNA might contribute to immunodeficiency, possibly leading to autoimmune diseases. Hence, such alterations could prove useful in diagnosing the disease and monitoring its progression. Furthermore, changes particularly pertaining to breast milk might affect breastfed newborns. Thus, the need to evaluate potential implications becomes apparent. The proposed mechanisms are visualized in **Figure 1**, which presents the potential impact of SLE-associated miRNAs on the development of the neonatal immune system.

The hypothetical model explores the potential implications of altered miRNA regulation in the bodily fluids of women with SLE, which may be associated with abnormal miRNA levels in breast milk. Milk-derived exosomal miRNAs could be absorbed by the neonatal GI system. As a result, dysregulation of miRNAs necessary for the development of a breastfed neonate, either through an insufficient or excessive supply, might compromise the infant’s immune response, possibly through pathways similar to those involved in SLE. In the absence of studies evaluating miRNA levels in the breast milk from mothers with SLE, this figure presents a hypothetical mechanism based on indirect evidence from SLE, breast milk, and neonatal studies. Furthermore, the level of evidence is visually represented using a color-coded scale. A blue background indicates direct evidence, whereas a white background represents hypothetical links. Due to the substantial number of sources suggesting the involvement of miRNAs in SLE and the limited evidence regarding their content in breast milk or their effect on newborns, only selected molecules are presented in **Table 1** (22–53). They were chosen based on the number of sources recognizing their influence in SLE, their presence in breast milk and their reported impact on neonates, which is discussed in the individual sections on each molecule.

**Table 1:** Reported changes in miRNA levels in patients with SLE: evidence from tissues and bodily fluids other than breast milk, with no neonatal evidence.

miRNA	Source material and study population [no. of healthy controls/patients with SLE]	miRNA level change in patients with SLE compared with healthy controls	Ref. no.
miR-155	serum [30/40]; [30/30]	down	(22, 23)
	serum [75/24]	up	(24)
	PBMC [40/60]		(25)
	urinary sediment [13/40]		(26)
	whole blood [32/40]		(27)
miR-146a	serum [30/40]	down	(22)
	serum exosomes [10/10]		(28)
	plasma [40/88]		(29)
	serum [10/20]; [39/42]; [40/40]	up	(30–32)
	whole blood [32/40]		(27)
	leukocytes [39/101]		(33)
	urinary supernatant [30/40]		(22)
	urinary sediment [13/40]		(26)
miR-21	serum [40/40]	up	(32)
	CD4^+^ T-cells [30/45]		(34)
	PBMC [25/34]; [8/20]		(35, 36)
	plasma [30/70]; [100/200]; [36/44]; [40/40]		(37–40)
miR-181a	T-cells [28/24]	down	(41)
	serum [75/24]; [70/67]; [40/100]	up	(24, 42, 43)
	plasma [30/70]		(37)
miR-98	CD4^+^ T-cells [39/48]	down	(44)
	PBMC [20/41]; [30/30]		(45, 46)
miR-210	PBMC [35/35]	up	(47)
miR-125b	PBMC [12/12]; [35/35]	down	(48, 49)
	neutrophils and monocytes [56/64]		(50)
miR-29a	serum [75/24]	up	(24)
	B-cells [10/66]	down	(51)

Only patients with SLE were included; cases of lupus nephritis were excluded. All the molecules in **Table 1** were analyzed using RT-qPCR.

All the selected miRNAs, mentioned in **Table 1**, have been detected in breast milk (5, 6, 15, 20, 52–54). However, their exact levels in breast milk remain unknown, as no standardized method for reporting results has been established. Thus, no reliable comparisons can be made between changes in miRNA levels in breast milk and in bodily fluids from patients with SLE. Importantly, to date there is no evidence in the scientific literature concerning miRNA levels in the breast milk from mothers with SLE. Furthermore, some studies addressing miRNAs as biomarkers of SLE present heterogeneous findings regarding changes in the levels of particular miRNAs in the same source material. Therefore, no conclusion can be drawn regarding possible patterns of alteration. Given the large number of dysregulated miRNAs in SLE, this review focuses on miR-155, miR-146a, miR-21, and miR-181a, for which substantial evidence supports a role in neonatal development. Other molecules listed in **Table 1** are addressed only briefly.

### 5.1 miR-155

As shown in **Table 1**, evidence regarding its dysregulation in patients with SLE has been obtained from various source materials, with contradictory results reported for plasma. The measurable changes in expression suggest its involvement in disease pathogenesis. miR-155 is believed to contribute to immune regulation through the control of genes involved in B-cell development, for instance activation-induced deaminase (AID) (55). In regard to SLE, one of its pathogenic mechanisms, associated with the impairment of Treg cells, is linked to miR-155/SOCS1 signaling axis. Suppressor of cytokine signaling 1 (SOCS1) is a target of miR-155 and regulates the stability of Treg cells (55). Notably, research has demonstrated that haploinsufficiency of one of the *SOCS1* genes is associated with early-onset autoimmunity (55). In support of this pathogenic mechanism in SLE, a study by Yu et al. demonstrated elevated expression of miR-155 along with decreased expression of *SOCS1* in Treg cells of patients with SLE. Their research also found a connection between the miR-155/SOCS and NF-κB signaling which is linked to inflammatory processes (56). Furthermore, Yu et al. explored the influence of miR-155 inhibition, which resulted in increased levels of CTLA4, LAG-3, TGF-β, and IL-10 under inflammation-like conditions (56). Cytotoxic T-lymphocyte-associated antigen-4 (CTLA4) is expressed on activated T-cells and reduces T-cell activation. For instance, its increased presence may be associated with atopic dermatitis (57).

From a developmental perspective, miR-155 is considered to modulate atopic sensitization in newborns. Inhibition of *SOCS1* by miR-155 enhances *FOXP3* expression, thus promoting Treg cells maturation. As a result, it reduces the Th2-mediated IgE-driven atopic immune response. The above-mentioned process can be decomposed into a sequence of events. Inhibition of *SOCS1* promotes STAT5 in the IL-2/STAT5 pathway, through TCR activation and CD25 upregulation. In addition, STAT5 together with SMAD5, which was previously activated by TGF-β, promotes *FOXP3*, which is responsible for Treg cells maturation. Moreover, miR-155 suppresses the mRNA of GATA3, a transcription factor involved in the atopic immune response, IL-4, a key cytokine in the Th2-mediated response, and PU.1, a transcription factor regulating immunoglobulin switching in plasma cells (57). Fresh cow milk, as well as maternal lipopolysaccharide (LPS)-mediated breast milk, has been shown to be a source of exosome-derived miR-155 (57). Notably, research has shown homology between human and bovine miR-155 (55). Therefore, breast milk of an atopic mother might contain reduced levels of miR-155 and contribute to the transmission of atopic diseases (57). Of note, there are speculations that high exposure to miR-155 in bovine milk might promote lymphomagenesis in elderly people (58). Intriguingly, miR-155 along with miR-21 was downregulated in mothers with hypertension (59). A study by Abaturov et al. presented preliminary experimental data suggesting that miR-155 may lower the risk of necrotizing enterocolitis in breastfed preterm infants which was further associated with decreased *FOXP3* levels in mucosal cells in the formula-fed group. Moreover, the breast milk of mothers delivering preterm was shown to contain increased levels of miR-155, which might suggest a role of miR-155 together with *FOXP3* in the prevention of necrotizing enterocolitis (60). Collectively, the significance of miR-155 can be observed both in SLE and in neonatal development. However, this topic is not yet fully elucidated and remains largely hypothesis-based.

### 5.2 miR-146a

Similarly to miR-155, miR-146a has been assessed in numerous studies in terms of its role in SLE pathogenesis. However, some of the results have led to conflicting conclusions concerning its dysregulation patterns. Multiple studies have examined miR-146a from a symptomatic perspective. For instance, decreased miR-146a expression may be associated with secondary antiphospholipid syndrome in patients with SLE or with ocular manifestations such as photosensitivity, cataract, dry eye, keratitis or drusen, whereas increased expression has been associated with retinopathy (2, 31). Some researchers attribute its symptomatology to cytokine expression. miR-146a regulates the immune response through Toll-like receptor (TLR) signaling pathway, in particular by targeting *IRAK1* and *TRAF6*. TLR signaling activates the IRAK1/TRAF6/IKKβ/NF-κB pathway axis which results in the expression of pro-inflammatory cytokines (61). Based on the research of El-Akhras et al., some manifestations of SLE were linked to the activity of cytokines such as IL-1β, IL-6 or IL-8 (61). In the context of miR-146a dysregulation in SLE, it is also associated with the disruption of type I IFN signaling pathway (62). This axis transduces signals through JAK/STAT and is linked to the immune response against viral infections (63). Of note, reduced miR-146a levels were suggested to be reversible by cyclophosphamide treatment, which may serve as a factor affecting the observed alteration patterns (61).

With respect to neonatal development, studies mainly point to the influence of miR-146a on the GI tract. In a bovine model, it was shown to contribute to the development of the GI tract (64). A study by Ghorab et al. reported that miR-146a was negatively correlated with inflammatory bowel disease activity as well as with inflammatory markers such as fecal calprotectin, CRP, and ESR in humans. The authors attributed the inflammation to an abnormal response to the microbiota caused by miR-146a deficiency and subsequent dysregulation of the NF-κB, IL-1, TLR and MyD88- dependent pathways along with type 2 macrophage polarization (65). According to Vahkal et al., inflammation in intestinal cells was attenuated by exosomes in breast milk. Based on their previous work, they proposed miR-146a-5p as one of the possible regulators of inflammasome activity and epithelium integrity in the intestine, as it was abundant in both term and preterm breast milk (66). Notably, a disease entity, such as necrotizing enterocolitis, was linked to JAK2 activity. Since this kinase is present in the JAK2/STAT signaling pathway activated by MYD88, it was suggested that miRNAs targeting both of those molecules might modulate inflammatory processes (66). Moreover, miR-146a levels were linked to asthma in children. Its upregulation was associated with an increased eosinophil percentage and forced expiratory volume in the first second (FEV1) (67). Interestingly, one study linked downregulation of miR-146a in breast milk to autism spectrum disorder in children (68). Despite the possibility that the aforementioned mechanisms may overlap in both SLE pathogenesis and infant development, the available evidence is insufficient to confidently describe the link between those areas of research.

### 5.3 miR-21

Similarly, miR-21 expression has been examined in various source materials. In contrast to the previously discussed miRNAs, the studies showed no conflicting results. Notably, Suo et al. linked overexpression of this molecule to a decreased level of complement component 3; however, they found no association with clinical manifestations (34). Stagakis et al. further investigated the mechanism of the disease, within the scope of miR-21 activity. Since patients with SLE exhibited overexpression of miR-21 in T-cells, their study further showed that inhibition of miR-21 led to decreased activation-induced proliferation of T-cells (35). As upregulation of miR-21 was also present in B-cells of patients, the study additionally demonstrated a reduction in factors possibly contributing to B-cell hyperactivity, namely IL-10 production and CD40L expression on the T-cell surface, in the absence of miR-21. Moreover, silencing of miR-21 led to a reduction in plasma cells and total IgG concentration (35). Overall, their research concluded that dysregulation of miR-21 might lead to immune dysfunction associated with SLE onset. In addition, a study by Ruan et al. showed that miR-21 could also affect T-cell apoptosis through regulation of *Tipe2*, also known as tumor necrosis factor-α-induced protein 8-like 2 (TNFAIP8L2), a protein known to contribute to immune homeostasis (69). Interestingly, Stagakis et al. also reported that PDCD4 levels were inversely correlated with miR-21 in patients with SLE (35). PDCD4 hinders tumorigenesis through suppression of cell growth and induction of apoptosis. Its significance is mainly attributed to the oncogenic role of miR-21 overexpression (70). In terms of immune response, the correlation between PDCD4 and miR-21 was suggested to be part of the inflammatory process stimulated by LPS in the NF-κB signaling (71).

The role of miR-21 in the neonatal immune system has mostly been considered in the context of antenatal development. Some studies link miR-21 expression in the placenta and maternal plasma to fetal growth (72–75). Of note, a study in human subjects showed that its upregulation in placental tissues might be linked to macrosomia (76). In addition, upregulation of miR-21 together with downregulation of NF-κB, TNF-α, and IL-6 mRNA levels was associated with possible infection-induced preterm birth in a mouse model (77). NF-κB signaling and its subsequent cytokine expression were examined as factors leading to preterm labor. Their findings showed that increased expression of miR-21 reduced NF-κB levels (77). Focusing on breast milk and atopy, one study measured miR-21-5p content in breast milk. However, it found no correlation between atopic disease in children and the intake of breast milk-derived miR-21-5p (78). On the other hand, studies have found a correlation between miR-21 and atopic diseases, such as atopic dermatitis, allergic rhinitis or asthma, associated with chronic lung diseases in infants (67, 79–82). However, some of those studies investigated the causes in the antenatal period. Intriguingly, Mielnik et al. also found high expression of miR-21 in Treg cells and, similarly to miR-155, described its connection to *FOXP3* (58). Thus, this may suggest a role in the atopic immune response. Lastly, miR-21-5p was reported to be influenced by the gut microbiota and, consequently, to be associated with the permeability of intestinal epithelium (83). Overall, most studies focus on antenatal development and suggest detrimental effects of miR-21-5p dysregulation on infant development. However, evidence concerning the development of breastfed children requires further research.

### 5.4 miR-181a

miR-181a has also attracted considerable research interest. In contrast to the previously discussed molecules, it has been studied in various populations, including Egyptian, Swedish, and Danish cohorts (37, 84). Additionally, a decrease in miR-181a level in PBMCs was observed in the pediatric population (85). Some studies have also suggested that specific genotypes may contribute to SLE risk (86, 87). With regard to SLE pathogenesis, miR-181a has been suggested to participate in B-cell differentiation, autophagy, regulation of inflammatory factors, and modulation of T-cell receptor sensitivity, thereby potentially hindering proper T-cell selection (12, 43, 88, 89). In particular, the differentiation of B- and T-cells was proposed to be stimulated by miR-181a/b through suppression of PTEN and subsequent promotion of PI3K/AKT signaling (89). Moreover, miR-181, through interaction with TGF-β and IL-10, was shown to physiologically affect the proliferation of Treg lymphocytes, thereby helping to prevent inflammatory processes (5).

miR-181a in breast milk is believed to play a crucial role in the development of oral tolerance in newborns. Moreover, a study carried out on humans and mice found that miR-181a influenced the expression of IL-10 and TGF-β in allergic rhinitis (5). Furthermore, in a porcine model, piglets whose neonatal diet consisted of dairy-based formula had decreased serum miR-181a levels compared with the group fed human milk (90). In addition, a study by Ahlberg et al. demonstrated a correlation between colostrum miR-181a-3p and the level of resting regulatory T-cells in breastfed infants of 6 months of age. miR-181a-3p levels in mature milk were also found to be linked with activated regulatory T-cells in infants of 24 months of age (91). In summary, miR-181a may prove to be important for the role of breast milk in child development.

### 5.5 let-7 family

Due to the limited availability of literature, the remaining miRNAs listed in **Table 1** are discussed only briefly. Nevertheless, their potential roles in developmental processes require further investigation. One such example is the let-7 family, which consists of 10 molecules: miR-98, miR-202, let-7a, -7b, -7c, -7d, -7e, -7f, -7g and -7i (92). The let-7 family also affects the immune response through TLR4 signaling pathway and activation of macrophages (90). One study demonstrated a suppressive effect of let-7 on TNFAIP3, a protein that suppresses NF-κB, thereby suggesting a link to processes such as inflammation and tumorigenesis (93). In SLE, miR-98 was thought to be related to dysregulated apoptosis and was found to be inversely correlated with IL-6 levels (44, 45). In terms of breast milk, let-7f-5p levels vary in allergic women, which might affect development of the newborn. For instance, the decreased let-7f-5p levels were associated with the development of atopic dermatitis in infants (5). In a porcine model, a reduction in let-7 family levels was noted in the group fed dairy-based formula (90). Additionally, a study by Ahlberg et al. showed a correlation between the colostrum let-7d-3p and the level of activated regulatory T-cells in infants of 24 months of age (91).

### 5.6 miR-210

Regulation of miR-210 is closely linked to hypoxia-inducible factors (HIF)-1α and -2α (2). In an SLE model involving both human and mouse cells, increased expression of miR-210-3p and HIF-1α was observed only in CD4+ T-cells. Of note, in lupus-prone mice this upregulation was positively correlated with increased disease activity (94). When considering its potential impact on breastfed offspring, one study showed increased expression of miR-210-5p in mammary epithelium of lactating mice exposed to cigarette smoke (95). Moreover, perinatal exposure was also found to increase miR-210 expression and possibly induce a hypoxic-ischemic brain-sensitive phenotype in neonatal rats (96). Another study associated upregulation of miR-210-5p in rats with early vascular dementia (97). Overall, these findings suggest that overexpression of miR-210 may be detrimental to neonatal development. However, it should be noted that all of the aforementioned studies were conducted in rodent models.

### 5.7 miR-125b

The role of miR-125b has been identified in the regulation of numerous signaling pathways, for instance NF-κB, p53, PI3K/Akt/mTOR, ErbB2 and Wnt (2). Interestingly, one study showed that miR-125b expression in patients with SLE was further reduced by exposure to UVB radiation, thus potentially suggesting a correlation with photosensitivity in these patients (48). As for breast milk-derived miR-125b, it may be significant in the regulation of GI cells in infants. Accordingly, it might also be related to the prevention of infantile bowel-associated diseases (98, 99). Notably, a study by Chiba et al. showed that the levels of miR-125b in the breast milk of mothers were reduced after 30 days postpartum (53).

### 5.8 miR-29a

In a group of patients with SLE, reduced miR-29a expression in B lymphocytes was linked to increased Crk-like protein expression resulting in enhanced IgG secretion (2). Increased levels of miR-29a-3p in breast milk were also correlated with a higher likelihood of infant colic, as miR-29a-3p has been proposed to downregulate tight junction proteins such as ZO-1 and claudin-1, thereby promoting inflammation in the GI tract (98).

## 6 lncRNAs in SLE and neonatal development

As stated above, lncRNAs refer to ncRNA molecules consisting of at least 200 nucleotides and may reach lengths of over 100 kb (100). They do not generally encode proteins; however, they may play important roles in cellular organization and regulation, for instance, in X-chromosome silencing. Hence, alterations in lncRNAs might contribute to the progression of numerous diseases, such as cancer, inflammation, or autoimmune disorders (2). Although lncRNA molecules are found in antisense transcripts among the protein-coding genes, they contain no open reading frame. They interact with genes located within 100 kb upstream or downstream through cis-regulation. For instance, regulation of *LMBRD2* may increase IFN-γ levels and possibly aggravate SLE activity (100). lncRNAs can bind to chromatin or DNA strands during transcription and mRNA splicing, link transcription factors to promoter and enhancer regions as well as bind to transcription factors themselves (100). Similarly to miRNAs, lncRNAs can be found in numerous bodily fluids (101).

Despite the presence of some lncRNAs in breast milk exosomes, only a limited number of studies have investigated their potential impact on breastfed infants (102) However, a study by Mourtzi et al. considered *NORAD* (non-coding RNA activated at DNA damage), an lncRNA regarded as a guardian of the genome due to its role in maintaining genomic stability. The study suggested that *NORAD* may be linked to the adaptive response of an infant to hypoxic conditions, as its upregulation in response to hypoxia was observed. Such conditions may, for instance, be encountered during the perinatal period. Hence, *NORAD* could potentially improve neonatal adaptation to oxidative stress. Of note, it was further explored that the level of *NORAD* was significantly downregulated in the breast milk of mothers who delivered preterm (102). Therefore, gaining insight into dysregulated lncRNAs in SLE may prove valuable in exploring the potential impact of breastfeeding on newborns. In comparison with miRNAs, the evidence regarding lncRNAs in breast milk and neonatal development is limited; thus, the section is addressed only briefly. Similarly to miRNAs, selected lncRNAs linked to both SLE pathogenesis and neonatal development are discussed below.

### 6.1 NEAT1

Patients with SLE were found to have upregulated *NEAT1* expression in peripheral blood mononuclear cells, which was negatively correlated with Th1/Th2 balance (2). Moreover, NEAT1 may potentially modulate the MAPK pathway, thereby affecting the inflammatory response in SLE (2). Research on *NEAT1* in terms of its impact on neonates and its transmission through breast milk is severely limited. A study by Karlsson et al. showed that *NEAT1* was present in exosomal vesicles in breast milk, although at a lower frequency compared with other studied lncRNAs (103). Additionally, its upregulation was observed in infantile pneumonia together with miR-146b (104).

### 6.2 TUG1

TUG1 is associated with the NF-κB pathway; thus, its role may be related to the inhibition of apoptosis and the production of inflammatory mediators. With regard to SLE, analysis of *TUG1* expression showed that it was downregulated in peripheral blood mononuclear cells from patients with SLE (2). Regarding breast milk, a study by Karlsson et al. showed that *TUG1* was detected in more than 50% of breast milk samples (103). *TUG1* has also been suggested to act as a mediator of proper neuronal development. Using a rodent model, one study demonstrated that *TUG1* expression was of great importance for the proper formation of photoreceptors in the neonatal retina (105).

### 6.3 GAS5

Since *GAS5* may mimic the glucocorticoid response, its function is associated with regulation of the androgen receptor, progesterone, and mineralocorticoid regulation (2). In patients with SLE, downregulation of *GAS5* in plasma has been reported (106, 107). However, another study reported increased *GAS5* expression in CD4+ T-cells (34). Of note, Suo et al. found a correlation between *GAS5* overexpression and ulceration in patients with SLE (34). *GAS5* was also found to be one of the most abundant lncRNAs in breast milk exosomes. Its activation has been associated with the response to growth arrest and cellular starvation caused by a lack of nutrients or growth factors. It has also been suggested to assist in programming the immune system of newborns (100). In accordance with this, a study by Karlsson et al. demonstrated that *GAS5* was present in 90-100% of breast milk samples (103).

## 7 Discussion

This review aimed to discuss the role of specific ncRNA molecules in the pathogenesis of SLE, as well as their potential involvement in infant development. Numerous studies have shown dysregulation of ncRNA expression during the course of the disease. Moreover, the question of whether ncRNAs in breast milk are crucial for the development of breastfed children is becoming increasingly important. However, the literature provides only limited knowledge on this topic. Most studies investigate dysregulation of ncRNAs in bodily fluids of patients with SLE, with the exception of breast milk. As a result, there is no direct evidence of altered ncRNA levels in the breast milk from mothers with SLE. Furthermore, the current evidence does not support discouraging breastfeeding in women with SLE. This area of research is highly relevant, as alterations in the ncRNA profile of breast milk from mothers with SLE might potentially influence the breastfed child. However, the current state of knowledge does not support definitive conclusions regarding the nature of the effect. Furthermore, the mechanism involved, as well as ncRNA molecules crucial to this process, have yet to be confidently and comprehensively described.

A vast number of miRNAs have been linked to the pathogenesis of SLE; therefore, this review focuses only on a selected few that are discussed in the largest number of sources related to SLE biomarkers and for which some information is available regarding their presence in breast milk, and their possible influence on the breastfed child. Nevertheless, most of the studies mentioned are based on single experiments and on the authors’ hypotheses. This review summarizes information on miRNAs, such as miR-155, miR-146a, miR-21, miR-181a, the let-7 family including miR-98, miR-210, miR-125b, and miR-29. However, studies on SLE have identified many more molecules, for instance miR-126, miR-132, miR-145, miR-23a-3p, or miR-4532 (23, 108). Apart from investigating the expression of miRNAs in bodily fluids of patients with SLE, many studies also expanded their scope to include, for example, SLE activity, usually assessed using the SLEDAI scale (SLE Disease Activity Index), or lupus nephritis. To illustrate, a study by Ibrahim et al. reported upregulation of serum miR-21 in patients with active disease, whereas miR-146a levels were higher in the inactive disease group, suggesting a negative correlation (32). With respect to lupus nephritis, results obtained by Khoshmirsafa et al. indicated that upregulation of miR-21 and miR-155 in PBMCs could serve as potential biomarkers of lupus nephritis (109). On the other hand, a study by Tangtanatakul et al. implied that decreased concentrations of let-7a and miR-21 in urine exosomes might serve as biomarkers of active lupus nephritis and be used for therapeutic monitoring (110). In addition, miR-146a was also considered a possible marker of lupus nephritis, especially since it was correlated with creatinine levels and disease activity (30).

Knowledge regarding miRNA biomarkers in SLE and their relationship to treatment is crucial, as it not only broadens future perspectives but also introduces another potentially confounding factor in analyses of miRNA composition, which was omitted in some studies. Although the evidence regarding the influence of SLE treatment on ncRNA composition is limited, some studies suggest a correlation between those variables. With respect to the future use of miRNAs as SLE biomarkers, they could potentially be utilized in targeted therapy. To illustrate, one study described a possible therapeutic mechanism involving umbilical cord mesenchymal stem cells (UC-MSCs) which downregulated inflammatory mediators. However, the authors also suggested that UC-MSCs might increase miR-181a levels in T-cells (41). In addition, another study showed that the expression of miR-155 and miR-146a increased after calcitriol treatment, with changes in miR-146a being directly linked to changes in the calcium-phosphate product (22). Moreover, glucocorticoid or hydroxychloroquine treatment was shown to increase miR-451a levels in CD 4+ T-cells of patients with SLE, whereas belimumab treatment resulted in upregulation of miR-146b-5p, miR-146a-5p, miR-125a-5p, and miR-29a-3p in T-cells of patients with SLE (111, 112). Thus, SLE treatment might affect the overall miRNA composition in bodily fluids and should be considered an important variable.

The concentration of miRNAs in breast milk may also depend on maternal health, which introduces potential confounding factors into these hypotheses. The composition of breast milk may change across lactation stages, as shown in studies by Raymond et al. and Verma et al. (113, 114) However, Alsaweed et al. identified several miRNAs that were present in each lactation stage in their study, including let-7f-5p, miR-181a-5p, miR-146b-5p, and miR-21-5p (115). Furthermore, delivery mode and gestational age are important determinants of breast milk miRNA composition (116, 117). Maternal diet and body weight may also influence miRNA levels. Overweight or obese mothers were studied by Shah et al., who showed that these women had reduced levels of miR-148a and miR-30b content at 1 month of lactation; however, after 3 months postpartum, no difference was noted. A positive correlation between miR-30b and infant weight was also observed, whereas miR-148a showed a negative correlation, mainly during the first month (118). Certain maternal diseases have also been found to affect miRNA content. For example, type 1 diabetes in mothers was associated with overexpression of miR-4497 and miR-133a-3p, as well as downregulation of miR-518e-3p and miR-200c-5p (119). Breast milk from women with hypertension was found to contain reduced levels of miR-126, miR-155, miR-21, and miR-29a (60). Dysregulation of miR-1290 was identified in mothers with active asthma and atopy during pregnancy. Mothers with active asthma alone during pregnancy were found to have downregulated levels of miR-30a-3p and miR-200a-3p (120). Inflammatory bowel disease has also been studied in terms of its impact on breast milk. According to Golan-Gerstl et al., miR-21 and miR-320 levels were reduced, whereas let-7a levels were increased. They also examined anti-TNF treatment in pregnant women, which was associated with reduced levels of miR-21 and miR-148a (121). In addition, Wang et al. reported increased levels of miR-146b in breast milk after epidural labor analgesia (122). According to Holzhausen et al., other important factors include postpartum day, the time of breast milk collection, predominant breastfeeding, and breastfeeding frequency (123). Overall, numerous maternal factors should be considered in future research on ncRNA composition.

With regard to lncRNAs, their involvement in SLE pathogenesis continues to be explored by numerous researchers. Apart from hypotheses concerning pathogenic mechanisms, lncRNAs may also contribute to sex bias of the disease through lncRNA *Xist*, which is involved in X chromosome methylation. Some of the molecules most frequently discussed in terms of their link to SLE include *NEAT1*, *TUG1* or *GAS5*. However, as this topic is currently receiving considerable attention in SLE research, many more molecules are being identified. A study by Liu et al. published in March 2025, identified 419 dysregulated lncRNAs in PBMCs. For example, 241 were shown to be upregulated, including LNC_005556 and LNC_008045, whereas 178 lncRNAs were downregulated, such as LNC_000099 and LNC_000127 (100). A study by Wang et al. focused on measuring of lncRNAs in monocyte-derived dendritic cells and identified 163 differentially expressed molecules in SLE. They emphasized the potential role of ENST00000604411.1 and ENST00000501122.2 in SLE activity (124). lncRNAs have also been identified as emerging mediators in cancer and have been discussed in terms of their role in carcinogenesis (125). However, the amount of knowledge regarding their content in breast milk and their impact on infant health remains substantially limited. Research by Zhou et al. suggested that upregulated lncRNA *MALAT1* might be linked to preeclampsia during pregnancy. They demonstrated that *MALAT1* had a target site for miR-144; hence, downregulation of miR-144 might be related to upregulation of this lncRNA. It is important to note that both molecules were measured in plasma (126). Qin et al. explored the role of lncRNA in hypoxia and hematopoiesis. They concluded that activation of HIF-1β under hypoxic conditions was correlated with downregulation of two particular lncRNAs - NONMMUT044528.2 and NONMMUT053442.2. Hence, they proposed a potential involvement of lncRNAs in fetal hematopoiesis and their contribution to hematopoietic disorders (127). Lastly, the work by Hussey et al. focused on the lncRNA content in placental tissue in relation to maternal pre-pregnancy BMI and infant birthweight in two cohorts. Their results demonstrated no overall correlations. Sex-specific differences were observed in the links between lncRNAs and birthweight, as they identified one transcript in the male population of one cohort and 17 transcripts in the female population of one cohort that corresponded to birthweight (128). Nevertheless, all of these findings and hypotheses remain to be verified through further analyses. Similarly, the topic of lncRNA levels in breast milk requires further investigation.

Concentrating specifically on allergic diseases and breastfeeding, there is insufficient evidence to confidently determine whether breastfeeding definitely suppresses their development (129). Nevertheless, multiple studies have linked specific miRNAs to allergic diseases. For example, Soujalehto et al. reported that levels of miR-205, miR-155, and miR-498 in the nasal mucosa were elevated in patients with current allergic rhinitis. Furthermore, let-7e was downregulated in patients with non-current symptoms of allergic rhinitis as well as asthma (130). Shifting the focus to atopic dermatitis, a study by Sousa et al. showed upregulation of, among others, miR-146a, miR-181b-5p, and let-7i-5p by IgG from patients affected by the disease. The authors also found a similarity in the upregulation of miR-92a, as it was shown to be increased in the nasal mucosa of patients with allergic rhinitis, in the plasma of patients with SLE, and ultimately, dysregulated in thymocytes in their experiment (131). According to a study by Hicks et al., infants consuming miR-375-3p contained in breast milk during the first 6 months of life were shown to have a lower likelihood of developing atopic dermatitis, wheezing, or food allergies in the first year (78). Nonetheless, a retrospective study by Lin et al. concluded that no particular pattern could be identified between atopic dermatitis and breastfeeding due to the heterogeneity of their results (132). Lastly, another group of researchers proposed, based on their findings, that miR-21, miR-155, miR-29a, miR-31 and miR-146a might be related to late-onset sepsis in neonates. They reported increased levels of miR-21 and miR-155 and reduced levels of miR-29a, miR-31 and miR-146a, with discrepancies in the data introduced by pre-term births, low birthweight, and non-survival (133).

This question regarding the impact of maternal SLE on offspring is heavily reliant on hypotheses and speculations, as the available literature is severely limited. Consequently, the compiled findings require rigorous verification in terms of their strength. The major gap in current knowledge is the lack of information on ncRNA levels in the breast milk from mothers with SLE. Direct evidence demonstrates that changes in ncRNA composition occur in the bodily fluids of patients with SLE. However, there are currently no reported findings regarding changes in ncRNA levels in the breast milk from mothers with SLE. Although this review explores the hypothesis that similar changes may occur, studies have presented differing expression profiles between breast milk and maternal circulation; thereby, suggesting endogenous production of ncRNAs in lactocytes. As a result, the hypothesis regarding ncRNA levels in breast milk of patients with SLE remains constrained by limited data availability. Of note, findings concerning ncRNA changes in bodily fluids remain inconclusive, as expression levels vary between studies. This observation may be attributable to non-uniform methodology, as well as to SLE treatment, disease manifestations, or disease activity. With regard to measurements of ncRNA content in breast milk, Holzhausen et al. noted that milk processing and EV isolation could affect the results (123). Furthermore, the method of pasteurization, variation in ncRNA content across different milk fractions, the choice of miRNA and RNA isolation kits, methods of milk storage, and casein removal strategies were also shown to yield variable results (134–137). In addition, the maternal factors mentioned above should be taken into account in future investigations. A further key consideration concerns the transfer of ncRNA through breast milk. The available data provide indirect evidence of the absorption and distribution of exogenous miRNAs in animal models (138–141). Moreover, *in vitro* models showed uptake of miRNAs from bovine milk by Caco-2 and THP-1 human cells (142, 143). Supportive evidence also demonstrated an increase in bovine miRNAs in human plasma after consumption of bovine milk (144, 145). Nevertheless, a study by Auerbach et al. did not reproduce similar results (146). Further *in vitro* studies provided some evidence for absorption of human breast milk, including preterm milk (147, 148). Kakimoto et al. identified possible breastfeeding biomarkers in gastric contents during biopsies performed in a very small group of infants. Nonetheless, their study also yielded preliminary data regarding the presence of some miRNAs in the gastric content of adults (149). Notably, Kompaneets et al. suggested that breast milk IgG could hydrolyze some miRNAs, thereby introducing another potential confounding factor (150). In terms of miRNA bioactivity, a study by Liang et al. presented preliminary evidence regarding regulation of gene expression in transfected human intestinal epithelial cells by miR-22-3p (151). Finally, Xu et al. addressed the existing disparities in the evidence. According to their study, the mechanism of absorption should be investigated more thoroughly using labeled, rigorously selected exogenous miRNAs, whereas research on bioavailability requires better understanding of miRNA metabolism, including the role of microbiota and possible sites of utilization in the GI tract (4). Taken together, the current understanding of ncRNA transfer through breast milk requires further investigation, as it is derived largely from studies with small sample sizes and from the absence of clinical models. In the context of neonatal development, the available evidence emphasizes the beneficial effect of breastfeeding on the infant. Even though the presence of ncRNAs in breast milk has been documented, there is no direct evidence of their necessity for the breastfed infant. As a result, the hypotheses explored in this review are based on isolated findings and speculative assumptions.

To summarize, there is insufficient evidence to draw reliable conclusions or verify the hypotheses concerning this topic. Further investigation in this area is important, as it may have implications for understanding SLE pathogenesis as well as for the prevention and diagnosis of SLE through ncRNA biomarkers. Moreover, future studies may help determine whether specific ncRNAs have functional relevance and whether this knowledge could inform the development of infant formulas. Possible directions of future research include comparative profiling of breast milk ncRNAs in women with and without SLE. Furthermore, this could be complemented by longitudinal studies examining changes in ncRNA composition in relation to disease activity or treatment status. In addition, this field would benefit from methodological standardization in the assessment of ncRNAs in bodily fluids, including breast milk. Further insights into the topic could also be provided by mother-milk-infant paired cohort studies evaluating the transfer and biological activity of selected ncRNAs.

## 8 Conclusion

In conclusion, the available studies provide insufficient evidence to determine whether breast milk from mothers with SLE may influence the development of the infant immune system. However, SLE is not considered a contraindication to breastfeeding. Nevertheless, the limited number of publications on this particular topic indicates that further investigation is essential before its impact can be confidently excluded. Overall, several ncRNAs with potential relevance to neonatal development can be identified in breast milk. Therefore, this topic requires further investigation in order to reach reliable conclusions. The current evidence does not support discouraging breastfeeding in women with SLE.
